# Household Hints and Recipes

**Published:** 1883-07

**Authors:** 


### Household Hints & Recipes.

CEMENT FOR AQUARIA OR WATER-TANKS may be prepared by intimtaely mixing

| | |
|---|---|
| Sublimed sulphur,<br>Ammonium chloride, powdered,<br>Iron filings, fine and sifted,<br>Boiled linseed oil,<br>Barium sulphate. | of each equal parts. |

SOOT STAINS ON MARBLE.—For removing the stains caused by drippings from stove pipes, it is suggested to apply powdered French chalk thoroughly moistened with benzine. Keep it closely covered to prevent evaporation, and after five or six hours apply another similar layer, and so on until the stain disappears. Chloroform may be used in place of benzine, if the latter does not serve.

KUMYS.—

Cow's milk, half skimmed, 21 pints.

Water 6 pints.

Milk sugar, ¼ lb.

Grape sugar, ¼ lb.

Sodium bicarbonate, 1½ oz.

Sodium chloride, ½ oz.

Brewer's yeast, 1½ oz.

Mix them and place the mixture for several hours in a warm place, at a temperature of 30 C. (86° F.). Then cool, strain, and transfer to stout bottles, which must be kept in a cool place. The product does not keep long.

REMOVAL OF FAT-STAINS FROM PAPER.—Oil or fat-stains, even when old, may be removed from paper or similar fabrics by means of a mixture of benzol and magnesia. Calcined magnesia is mixed with a sufficient quantity of benzol to produce a mass which becomes granular after a while. A little of this mixture is rubbed with the finger upon the stain and the little granules of magnesia afterwards wiped off. Fresh stains usually disappear entirely, old ones after a short time, particularly if the treatment is repeated two or three times. A great advantage of this method, originally introduced by Hirzel, is this, that it may be used upon the finest kind of paper and that it scarcely affects printed paper; prolonged contact only rendering the printing paler.

The mixture should be preserved in well-closed glass-stoppered vials.—*Neueste Erfind. und Erfahr.*

POSITIVE COPIES OF DRAWINGS, ETC.—Suitable paper is coated with a two per cent. solution of bichromate of ammonium, to which a little grape sugar has been added, and dried in the dark. The paper containing the drawing is laid upon it and exposed to light until the prepared paper has assumed a gray color. It is now dipped into a one per cent. solution of nitrate of silver, one-tenth of the volume of which consists of acetic acid. The positive image developed thereby consists of bichromate of silver, which becomes dark brown on drying.—*Phot. News and Chem. Zeit.*

MARKING INK.—The following process which has recently been patented in Germany by Ch. Knab, furnishes a *deep blue marking ink* for marking packages, etc., which is said to completely resist the influence of weather, etc.

Fifty kilos of a liquid logwood extract (containing 30 per cent. of the dry) are mixed with 3 liters of alcohol, to which 1 kilo of hydrochloric acid had previously been added, and the mixture, which is to be constantly stirred, to be kept at a temperature of 20° C. Five kilos of chromate of potassium are dissolved in 15 liters of boiling water; 10 kilos of hydrochloric acid are added, and the mixture, after being heated to 30° C., *very* slowly added to the former, contained in a suitable boiler. The whole is now heated to 85° C., the extract-like mass well stirred, and finally mixed with 15 kilos of dextrin and 10 kilos of fine white bole. The mass is then transferred to a mill, where it is thoroughly worked together and finally transferred to tin boxes, in which it is allowed to dry.

MARKING INK FOR RUBBER STAMPS.—Adolph Vomaca recommends the following practical method, which replaces the marking pad and vial with ink.

Thirty to forty parts of concentrated glycerin are saturated with the desired color (blue, red, green, black, etc.), which must be an aniline color of the darkest corresponding tint, and soluble in water. Ten parts of glue are soaked in cold water for twenty-four hours, the excess of water then poured off, the swelled glue dried rapidly between towels and then melted on a water-bath in the above mentioned glycerin, of which a larger or smaller quantity is required, according to the quality of the glue. The excess of water is evaporated off, taking care not to stir the mass so as to incorporate air bubbles, and the mass is then poured into suitable receptacles.

If, on standing, any air bubbles show themselves, they may be removed with a stiff cardboard. The smooth surface of elastic glue now represents the color-pad, and yields up its color evenly and slowly. If the mass becomes uneven it may be restored by simply melting, and if it should become a little hard on the surface, it need only be rubbed over with a sponge dipped in hot water.

If metallic stamps are to be used upon these glue-pads, the smooth surface of the former should be roughened by fine emery or sand-paper.—*Rundschau f. d. Pharm.*

---

BLACKBOARD-PAINT OR VARNISH.—A good black-board should not only have a deep black surface, without gloss, but should also have a fine grain so as to readily take the marks made upon it by chalk.

Among the more recent methods for producing such a surface, the following may be quoted:

Dissolve 9 oz. of shellac and 2¼ oz. of sandarac in ½ gallon of alcohol, at a gentle heat; and also 1 oz. of guttapercha in 5½ fl. oz. of oil of turpentine. Mix both solutions together, then add 1 lb. of finely powdered emery and 1½ oz. of lampblack. Put a thick coat of this paint on the wood, then place the black-board in a horizontal position and set fire to the paint at the lower edge. When the alcohol has been burned off, extinguish the flame and afterwards apply a fresh coat. When dry, paint again four or five times, but without igniting the alcohol. If any of the first coats show uneven spots or blisters, they may be removed by careful sand-papering.

---

LIQUID GLUE.—Take of the best white glue, 8 parts. Cut it fine, cover it with distilled water and soak it until soft; then pour off any excess of water. Now melt it on the water-bath, and add to it 1 part of glacial acetic acid. When thoroughly mixed, bottle it.

Among other recipes, the following may be quoted:

A solution of 1 part of sugar in 3 parts of water is mixed with ¼ part of dry slaked lime (calcium hydrate), and the mixture, after being heated to near 165°F., allowed to macerate for a few days, being frequently stirred. Most of the lime will thus dissolve, and the syrupy solution will be found to have considerable adhesive properties. To every 15 parts of the solution 3 parts of glue, in small pieces are now added, and made to dissolve by gently warming. On cooling, the mass will remain liquid without losing its adhesiveness. This liquid glue may be used for a variety of purposes, but must not be used in presence of coloring matters which are affected by lime, such as Prussian-blue, zinc-green, etc.

Or, dissolve 100 parts of ordinary glue or gelatin in 400 parts of water, together with 7 parts of oxalic acid, by heating on a water-bath in a porcelain dish. After 5 or 6 hours' heating, neutralize the oxalic acid with chalk, dilute with a considerable amount of water, and cover the solution well or keep it in a bottle until the precipitate (or as much of it as possible) has settled. Then evaporate to 200 parts. This is said to yield a good, light-colored liquid glue.

---

HOW TO LACQUER BRASS.—A correspondent to the *Scientific American* communicates the following practical directions:

1. Be sure there is no oil or grease on the brass; do *not* touch the work with the fingers, hold it with spring tongs or a taper stick in some of the holes.
2. *Always* handle with a piece of clean cloth.
3. Heat the work so hot that the brush will smoke when applied, but avoid overheating, as it burns the lacquer.
4. It is well to fasten a small wire across the lacquer cup, from side to side, to scrape any superfluous lacquer. The brush should have the ends of the hairs all exactly even. If not so, trim the ends with sharp scissors.
5. Scrape the brush as dry as possible on the wire, making a flat smooth point at the same time.
6. Use the very tip of the brush to lacquer with, and carry a steady hand.
7. Put on at least two coats. It is well (to make a very durable coat), to "blaze off" after each coat, with a spirit lamp or Bunsen burner, taking care not to burn the lacquer.
8. If the lacquer is too thick, it will look gummy on the work. If too thin, it will show prismatic colors. In the first case, add a little alcohol, in the latter, set the cup on the stove and evaporate some.
9. A good deal of cheap work, like lamp burners, is "dipped." Use a bath of nitric and sulphuric acids, equal parts, dip work, hung on wire, into acid for a moment, remove, rinse in cold water thoroughly, dip in hot water, remove, and put in alcohol, rinse around, then dip momentarily in lacquer, shaking vigorously on removing to throw off extra lacquer and lay on a warm metal plate till dry, let cool and it is done.
10. Avoid handling lacquered work until *cold.*

